# Prevalence of Nutritional Risk and Obesity in Mexican Cancer Outpatients

**DOI:** 10.3390/jcm14051559

**Published:** 2025-02-26

**Authors:** Gabino Cervantes-Guevara, Blanca Ernestina Vázquez-López, Lisset Magaña-de la Vega, Fernanda Monserrat Rendón-Serrano, Clotilde Fuentes-Orozco, Alejandro González-Ojeda, José Alberto González-Duarte, Diana Mercedes Hernández-Corona, Tonatiuh González-Heredia, Miriam Méndez-del Villar, María Fernanda Isadora Meraz-Corona, Milton Omar Guzmán-Ornelas, Verónica Chávez-Tostado, Mariana Chávez-Tostado

**Affiliations:** 1 Department of Wellbeing and Sustainable Development, Northern University Center, University of Guadalajara, Colotlán 46200, Mexico; gacergue@gmail.com; 2 Health Sciences University Center, University of Guadalajara, Guadalajara 44410, Mexico; nutri.blancavazquez@gmail.com (B.E.V.-L.); lissma14@gmail.com (L.M.-d.l.V.); fernanda.rendon.serrano@gmail.com (F.M.R.-S.); 3 Biomedical Research Unit 02, Specialties Hospital, Western National Medical Center, Mexican Social Security Institute, Guadalajara 44340, Mexico; clotilde.fuentes@gmail.com (C.F.-O.); avygail5@gmail.com (A.G.-O.); 4 Colorectal Surgery Unit, Civil Hospital of Guadalajara “Fray Antonio Alcalde”, Guadalajara 44200, Mexico; dr.jagd@gmail.com; 5 Department of Biomedical Sciences, Health Sciences Division, Tonalá University Center, University of Guadalajara, Tonalá 45425, Mexico; dianahernandez19@gmail.com (D.M.H.-C.); drtonatiuhgh@live.com.mx (T.G.-H.); miriamendez@hotmail.com (M.M.-d.V.); mariaf.corona@academicos.udg.mx (M.F.I.M.-C.); milton.guzman@academicos.udg.mx (M.O.G.-O.); 6 Department of Reproduction, Health Sciences University Center, University of Guadalajara, Guadalajara 44410, Mexico

**Keywords:** malnutrition, cancer outpatients, nutritional screening, GLIM criteria

## Abstract

**Introduction:** Malnutrition is a critical issue among cancer patients, leading to adverse clinical outcomes, including increased treatment toxicity, reduced physical function, and decreased survival. Nutritional screening is essential to identify patients at risk and provide timely interventions. **Objectives:** This study aimed to assess the effectiveness of various nutritional screening tools in identifying the risk of malnutrition and obesity in Mexican cancer outpatients. **Methods:** A cross-sectional study was conducted with 396 adult cancer outpatients at a public hospital in Mexico. Nutritional risk was evaluated using NRS-2002, MUST, MST, NUTRISCORE, and PG-SGA, while malnutrition was assessed using GLIM criteria and PG-SGA. Anthropometric and demographic data were collected. Sensitivity, specificity, and kappa coefficients were calculated to determine the performance of the screening tools. **Results:** Nutritional risk was identified in 22.7–26.5% of patients, with the highest agreement observed between MUST and PG-SGA (k = 0.64). Malnutrition prevalence was higher using GLIM criteria (37.4%) compared to PG-SGA (25.8%, *p* < 0.001). Overweight and obesity affected 37.1% and 23.5% of patients, respectively. Low BMI and reduced HGS were strongly associated with nutritional risk and malnutrition (*p* < 0.001). **Conclusions:** MUST and PG-SGA are reliable tools for nutritional screening in cancer outpatients, while GLIM criteria detect a higher prevalence of malnutrition than PG-SGA. The high rates of overweight and obesity highlight the complex nutritional challenges in this population, emphasizing the need for tailored nutritional assessments and interventions.

## 1. Introduction

Cancer represents a significant burden, with morbidity and mortality rates rising across the globe [1]. Malnutrition is an unfortunate condition commonly observed in the oncology population [2,3,4,5] and is acknowledged to be an important prognostic factor [6], with a high prevalence between 30% and 80% [7,8,9,10,11]. Malnutrition during cancer is associated with several complications, including treatment toxicity, clinical complications, reduced physical function, longer hospital stays, and higher mortality rates [12,13,14], and is also associated with reduced handgrip strength (HGS) [15]. Therefore, it is crucial to identify and treat patients at nutritional risk during the early stages of the disease and throughout oncological treatment [16].

Nutritional screening (NS) aims to detect potential nutritional risks early so that a more detailed evaluation can be conducted if necessary. It is usually the first step in the nutritional care process and should be performed within the first 24 to 48 h of admission and repeated periodically, given the nutritional deterioration associated with prolonged hospitalization [17,18]. NS is often overlooked and limited by oncologists, caregivers, and health institutions [13,19,20,21,22], resulting in over 50% of patients at nutritional risk going unrecognized and only one-third of patients at nutritional risk receiving the nutritional support they need [6,22,23]. The European Society for Clinical Nutrition and Metabolism (ESPEN) recommends the following tests in cancer patients: Nutritional Risk Screening 2002 (NRS-2002), Malnutrition Universal Screening Tool (MUST), and the Malnutrition Screening Tool (MST) [8,24]. In addition, the Academy of Nutrition and Dietetics recommends the use of the MST and MUST [5] in cancer patients. Recently, the NUTRISCORE test, a novel nutritional screening tool designed for once-hematologic outpatients, included questions regarding cancer sites and active treatment and was now validated by ESPEN [25].

The Patient-Generated Subjective Global Assessment (PG-SGA) is considered the gold standard for detecting malnutrition and/or malnutrition risk in cancer patients. It is supported by ESPEN and the Oncology Nutrition Dietetic Practice Group of the American Dietetic Association [2,5,8,26,27]. It has demonstrated high sensitivity and specificity for nutritional assessment in oncology settings but not for nutritional screening [28,29,30,31,32,33]. Interestingly, the Global Leadership Initiative on Malnutrition (GLIM) has recently put forth diagnostic criteria to standardize clinical practices in diagnosing malnutrition within clinical settings by incorporating the assessment of disease burden and inflammation, providing a more comprehensive approach to diagnosing malnutrition. The validation of the GLIM criteria for the identification of malnutrition within an ambulatory oncology population, as well as their predictive value concerning patient survival, has not been established yet, as only a few studies have been conducted in the oncology population using these criteria [34]. The aim of this study was to evaluate the performance of different nutritional screening tools in identifying nutritional risk and their association with malnutrition, overweight, obesity, and HGS in Mexican cancer outpatients and to compare the prevalence of malnutrition identified by the GLIM criteria and the PG-SGA in this population.

## 2. Materials and Methods

An observational cross-sectional study of adult patients receiving ambulatory anticancer treatment during 2019–2023 was conducted at Hospital “Fray Antonio Alcalde” in Guadalajara, Mexico. Data collection and measurements were performed during medical follow-up of patients in the oncologic ambulatory clinic by an accredited dietitian.

Participants: Inclusion criteria were as follows: a positive diagnosis of cancer, age 18 years or older, ability to complete the process of nutritional screening and hand grip strength, and willingness to participate in the study. Patients were excluded from the study if they were receiving hospice treatment, lacked the capacity to understand the purpose of the study, or had severe functional impairment of vital organs or life-threatening conditions that could compromise safe participation, including advanced heart failure, uncontrolled arrhythmias, severe respiratory failure, advanced chronic obstructive pulmonary disease, pulmonary fibrosis, end-stage kidney disease requiring dialysis, severe hepatic failure or cirrhosis, and severe neurological disorders like advanced dementia or neurodegenerative diseases. Those with organ failure or severe infections due to multiple surgeries were also excluded. These criteria were established to ensure the safety and appropriateness of the study’s participants, as well as to protect their rights and welfare. Prior to participation, all participants were fully informed about the study’s objectives upon admission.

Clinical data: All data were collected from the patient’s electronic health record and during medical follow-up. This included anthropometric data such as height, weight, weight loss percentage in the previous month and 6 months, body mass index (BMI), age, current treatment, consumption of nutritional supplements, and HGS. Body weight was determined using a TANITA BC 601^®^ scale (Tanita Corporation, Tokyo, Japan). HGS was measured using a Jamar hand dynamometer^®^ (Sammons Preston Inc., Bolingbrook, IL, USA); the measurements were conducted with the patient standing and with the elbow and wrist fully extended. The measurements of both hands were taken twice at 5 s intervals, and the means were recorded. HGS was defined as the maximally measured grip strength of the dominant hand and was considered reduced for values < 16 kg in females and <27 kg in males [35].

GLIM criteria: The GLIM criteria represent a two-step model for the detection and diagnosis of malnutrition. The initial step in the process is nutritional screening, conducted using a validated screening test. The second step is to detect phenotypic criteria (non-volitional weight loss, low BMI, and reduced muscle mass) and etiologic criteria (reduced food intake or assimilation and disease burden/inflammation) in patients with nutritional risk [34]. Since cancer meets the etiological criterion of the GLIM criteria, patients who meet one of the three phenotypic criteria (non-volitional weight loss > 5% within the past 6 months or >10% beyond 6 months, low BMI < 20 kg/m^2^ for patients aged < 70 years or <22 kg/m^2^ in patients aged ≥ 70 years, and reduced muscle mass) were diagnosed with malnutrition. The remaining participants were categorized as well-nourished. PG-SGA: The PG-SGA was derived from the Subjective Global Assessment (SGA) for the oncology population and is considered the gold standard for this population [2,5,8,26,27]. This assessment test permits the classification of patients into three categories: well-nourished (PG-SGA “A”), moderately nourished with suspected risk for malnutrition (PG-SGA “B”), or severely malnourished (PG-SGA “C”). For data analysis purposes, malnutrition was assumed with a score of B or C. The remaining participants were classified as well-nourished. Nutritional Screening: Following established guidelines [24], all participants were screened for malnutrition using the NRS-2002, MUST, MST, and NUTRISCORE. All patients were reclassified as “at nutritional risk” if they had a score of ≥3 by the NRS-2002, a score ≥ 2 by the MST, ≥1 according to MUST, and >5 with NUTRISCORE.

Statistical analysis: Categorical variables are expressed as percentages and crude numbers, while continuous variables are expressed as the mean ± standard deviation (SD). Analysis was performed using Student’s *t*-test or the Mann–Whitney nonparametric U test for quantitative data, and the chi-square test or Fisher’s exact test for qualitative data. Statistical significance was defined as *p* < 0.05 (two-tailed). Statistical analyses were conducted using Excel 2020 (Microsoft, Redmond, WA, USA) and IBM SPSS Statistics software (version 20 for Windows; IBM Corp., Armonk, NY, USA). GLIM was compared with nutritional screening tests using the reference instrument (PG-SGA). Cohen’s kappa coefficient (k) was calculated to measure the agreement between all tests, with 95% confidence intervals (CI). The Shrout classification [36] was used to interpret values as follows: 0–0.1, virtually none; 0.11–0.4, slight agreement; 0.41–0.6, fair agreement; 0.61–0.8, moderate agreement; and 0.81–1, substantial agreement.

## 3. Results

### 3.1. Demographic and Clinical Data

The study sample included 396 patients, 72% of whom were female. The distribution of tumor locations in the sample showed that breast cancer was the most prevalent, accounting for 39.2% of cases, followed by colon and rectum cancer at 32.7%. Among the sample, 52.0% were undergoing chemotherapy, while 39.4% were not receiving any treatment. The mean BMI was 26.2 ± 5 kg/m^2^. Only 5.3% of patients were classified as undernourished, whereas 37.1% were overweight, and 23.5% had obesity.

During the last month, a total of 168 individuals (42.4%) experienced weight loss. Among them, 147 individuals (37.1%) had moderate weight loss (<5%), while 21 individuals (5.3%) experienced severe weight loss (>5%).

Among all patients with weight loss, 64.3% had obesity or overweight, and a moderate weight loss was observed in 67.3% of them. A total of 245 patients (62.0% of the total) were receiving treatment. Among them, 134 patients (56.1% of the treatment group) had obesity or overweight, compared to 105 patients (43.9% of the non-treatment group) with obesity or overweight. This difference was statistically significant (*p* = 0.003). The mean HGS was 24.2 ± 8.4 kg, with men showing significantly higher values than women (33.4 ± 8.01 kg vs. 20.6 ± 5.2 kg, respectively; *p* < 0.001). Low HGS was observed in 21.2% of patients, of whom 64.3% were female and 35.7% were male.

Among patients without obesity, 36.5% had breast cancer, while 41.0% of patients with obesity or overweight were diagnosed with breast cancer. Similarly, colon and rectum cancer was present in 30.8% of patients without obesity and 33.9% of those with obesity or overweight. Tumors in the head and neck were rare, representing only 3.0% of the total, with an equal distribution between the two groups. Liver, pancreas, and biliary tract tumors were slightly more frequent in patients without obesity (7.7%) compared to those with obesity or overweight (2.5%), while gastric tumors showed a balanced distribution of 5.8% and 3.8%, respectively. Tumors classified as “other” accounted for 15.9% of the total, with similar proportions in both groups. No statistically significant differences were observed in tumor location between patients with and without obesity or overweight. All results are shown in Table 1.

Antineoplastic treatment was administered to 67.3% of patients with moderate weight loss, compared to 32.7% of patients without treatment who also experienced moderate weight loss (*p* = 0.028; 95% CI: 0.36, 0.14–0.92).

Among all patients with weight loss, 8.9% were non-obese and did not receive treatment, whereas 26.7% were non-obese and received treatment. In contrast, 26.5% of patients with obesity/overweight were untreated, while 33.8% had obesity/overweight and received treatment. This association was statistically significant (*p* < 0.001).

### 3.2. Risk of Malnutrition and Malnutrition

The presence of malnutrition was identified in 25.8% of patients according to the PG-SGA, whereas the GLIM criteria detected malnutrition in 37.4% of patients (*p* < 0.001), indicating moderate agreement (κ = 0.63). Regarding nutritional screening tools, NRS-2002 identified risk in 22.7% of patients, NUTRISCORE in 12.9%, MUST in 25.8%, and MST in 26.5%. The PG-SGA and MUST demonstrated moderate agreement (κ = 0.64), while the GLIM criteria and MUST showed similar moderate agreement (κ = 0.68). The agreements observed among the other nutritional screening tests ranged from slight to fair. The degree of concordance and the associated outcomes across the different screening tools are summarized in Table 2.

Patients without obesity were consistently identified as being at higher risk of malnutrition compared to those with obesity across various nutritional screening tools. The GLIM criteria detected malnutrition in 49.4% of patients without obesity versus 29.7% of those with obesity (*p* < 0.001).

Similarly, other tools, such as PG-SGA, NRS-2002, MUST, and NUTRISCORE, reported higher malnutrition risk among patients without obesity, with statistically significant differences (*p* < 0.05). However, the MST showed no significant difference in risk between the groups.

These results highlight the potential limitations of standard nutritional screening tools in accurately assessing malnutrition risk in patients with obesity or overweight, likely due to differences in body composition and clinical presentation. The results can be observed in Table 3.

## 4. Discussion

Our study revealed that the identification of nutritional risk varies depending on the screening tool utilized in cancer outpatients. Using the NRS-2002, 22.7% of patients were identified as being at nutritional risk, compared to 12.9% with NUTRISCORE, 25.8% with MUST, and 26.5% with MST. The highest agreement was observed between MUST and PG-SGA (κ = 0.64) and between GLIM criteria and MUST (κ = 0.68). These findings suggest that these tools can be effectively used in combination to provide a comprehensive assessment of nutritional status and risk. Similarly, Gascón-Ruiz et al. [37] reported comparable agreement between the GLIM criteria and MUST (κ = 0.66) in an observational cross-sectional study involving 165 cancer outpatients. In this study, MUST demonstrate high specificity and sensitivity in detecting malnutrition. Additionally, Bozzetti et al. [6] observed that 32% of cancer outpatients were identified as being at nutritional risk using the NRS-2002. Moreover, a multicenter cross-sectional study involving 1000 cancer patients further highlighted discrepancies among screening tools [38]. When malnutrition was assessed using PG-SGA, NUTRISCORE, and MST, the prevalence of malnutrition was 45.0% with PG-SGA, 2.9% with NUTRISCORE, and 36.7% with MST. The kappa coefficient for NUTRISCORE was 0.066, indicating slight agreement with PG-SGA, whereas MST demonstrated moderate agreement (κ = 0.262). These results align with our findings, where MST identified a higher prevalence of malnutrition compared to NUTRISCORE, with similar kappa values. In another study evaluating malnutrition among colorectal cancer patients [39], GLIM criteria identified 10–24% of patients as malnourished, compared to 15% identified by the PG-SGA short form (PG-SGA-SF). The agreement between PG-SGA-SF and GLIM criteria varied: minimal agreement was observed with MUST (κ = 0.28), weak agreement with MST (κ = 0.42) and the first four questions of NRS-2002 (κ = 0.49), and moderate agreement with PG-SGA-SF (κ = 0.60). In our study, we observed an overall agreement of κ = 0.63 between GLIM criteria and PG-SGA, consistent with previous research. This level of agreement indicates that, while discrepancies exist, the GLIM criteria represent a viable alternative to PG-SGA for diagnosing malnutrition in colorectal cancer patients, as demonstrated in the ongoing CRC-NORDIET study.

Regarding malnutrition, our results indicate that 37.4% of ambulatory cancer patients were diagnosed with malnutrition according to the GLIM criteria, compared to 25.8% with PG-SGA. The disparity we observed suggests that the GLIM criteria may identify more patients at risk of malnutrition than the PG-SGA, potentially due to its comprehensive approach that includes disease burden and inflammation [34]. The results are comparable to those of the study by Gascón-Ruiz et al., who investigated the impact of malnutrition on cancer patients using GLIM criteria and found a prevalence of malnutrition in 46.6% among 165 cancer outpatients [40]. Another study that aimed to assess the predictive power of the GLIM criteria for postoperative pulmonary complications in cancer patients found that 32.1% had malnutrition [41]. Okada G et al. also found that 44% of esophageal cancer patients were diagnosed with malnutrition based on the GLIM criteria; moreover, symptoms such as dysphagia and esophageal obstruction were significantly associated with the severity of malnutrition [42]. In relation to PG-SGA, similar results were described in the study by Arribas et al., where outpatient cancer patients were observed to have a risk for malnutrition in 19% of the patients with PG-SGA [43]. Additionally, Abbott J et al. found malnutrition in 17% of patients according to PG-SGA among chemotherapy outpatients. It demonstrated high sensitivity and specificity, making it a reliable method for early detection of malnutrition [2]. Other studies of chemotherapy outpatients have reported a malnutrition prevalence of around 25%, which is very similar to our results [44,45]. Despite the established consensus on the GLIM criteria among experts regarding the assessment of malnutrition in outpatient cancer patients, there remains a limited number of comparative studies evaluating their validity in conjunction with screening tools. Only five studies were identified utilizing the GLIM criteria [37,40,46,47,48]. Notably, Gascón et al. (2022) [40] reported a malnutrition prevalence of 53.3%, highlighting significant differences across various cancer types. Most of the reviewed studies employed the PG-SGA as either the diagnostic standard or as a screening tool to detect malnutrition risk [47,48,49,50,51]. It is worth noting that many studies assessing malnutrition rely on indicators such as BMI [5,50,52], which have important limitations such as recent weight loss, recent food intake in the past few days, etc. Future studies should focus on more accurate approaches, incorporating advanced tools like body composition analysis via BIA, assessments of muscle function, dynamometry, and measures of physical performance [53,54]. The GLIM criteria, in particular, show promise as a robust framework for evaluating nutritional status and predicting survival in older cancer patients [55].

In relation to the presence of obesity or overweight, we found them combined in 60.5% of patients, with 37.1% classified as overweight and 23.5% as obese. These results align with those of Sánchez-Migallón et al. (2023), who reported that 20.8% of their cohort were overweight and 16.7% were obese among oncology patients with an average age of 64.8 years [56]. Also, in the recent cohort study of Vaidya R et al. (2022) [57], they examined clinical treatment trials for obesity-related cancers conducted by the SWOG Cancer Research Network at community and academic sites. Among 23,926 cancer patients enrolled between 1986 and 2016, obesity rates increased from 23.5% to 42.3%. Moreover, the combined prevalence of obesity and overweight was approximately 54.2% around 1986, increasing to 75.3% by 2016, which is very similar to the results observed in our study. The discrepancies in prevalence rates between studies may be attributed to differences in patient demographics, methodologies, and criteria for assessing obesity and overweight, such as the use of BMI. Furthermore, these findings underscore the importance of addressing excess weight in oncology care, given its association with adverse outcomes, including increased risk of cancer recurrence, reduced quality of life, and comorbidities such as cardiovascular disease and diabetes mellitus. Our findings suggest the significant prevalence of obesity and overweight among oncology patients, emphasizing the growing need for targeted nutritional interventions in this population.

Research has consistently shown that between 30% and 70% of cancer patients experience moderate to severe weight loss [58,59,60]. We observed a prevalence of 42% of moderate to severe weight loss during the last month in our patients. These results are consistent with the findings of previous studies conducted over the past 35 years. However, the prevalence of weight loss in the last month was higher among those undergoing oncological treatment compared to those who were not undergoing such treatment. According to BMI, we observed that obesity or overweight is present in a significant proportion of the overall patient population (60.5%), with a total of 37.1% with overweight and 23.5% with obesity. Only 5.3% of patients were malnourished. Sánchez-Migallón et al. (2023), found in a cohort of 96 oncology patients with an average age of 64.8 years, where 20.8% were classified as overweight, and 16.7% were classified as with obesity. Also, in a German study by Loosen SH et al. in 2022, they assessed overweight and obesity in 287,357 cancer outpatients and found a prevalence of overweight in approximately 38.2% and obesity in 32.4% of patients (70.6% combined) [61]. These findings highlight the prevalence of weight issues among cancer patients, with data cross-referenced between clinical histories and referral service records. Also, we observed moderate weight loss, which was more common in patients with obesity or overweight, accounting for 67.3% of moderate cases and 58.9% of the total patients analyzed. These findings suggest that patients with obesity or overweight are more likely to experience weight loss than those without excess weight.

The findings of this study reveal significant differences in malnutrition risk detection based on the presence or absence of obesity or overweight, highlighting the challenges of nutritional screening in this population. Across multiple tools, patients without obesity consistently demonstrated higher malnutrition risk compared to those with obesity or overweight. For example, the GLIM criteria identified malnutrition in 49.4% of patients without obesity, nearly double the prevalence observed in patients with obesity (29.7%, *p* < 0.001). Similar trends were noted with the NRS-2002, MUST, PG-SGA, and NUTRISCORE, all of which showed a statistically significant lower risk of malnutrition in individuals with obesity or overweight. These findings may be influenced by the metabolic and physiological differences between individuals with and without obesity. Patients with higher body fat reserves might mask clinical indicators of malnutrition or present less visible weight loss, which is a key criterion in many screening tools. However, this does not necessarily reflect the absence of malnutrition but rather underscores the limitations of these tools in accurately assessing nutritional risk in patients with higher BMI values. However, the overall agreement among tools was variable, with some demonstrating only slight to moderate concordance. This underscores the need for a tailored approach in selecting and interpreting nutritional assessment tools, particularly in populations with obesity or overweight. Many cancer survivors tend to gain weight after their diagnosis and treatment [62,63]. Obesity not only heightens the likelihood of cancer recurrence in certain cases but also raises the risk of developing diabetes, cardiovascular diseases, and reduced quality of life. Implementing weight loss strategies is a crucial aspect of post-treatment care for cancer patients who are overweight [64,65].

Our study addresses a critical gap in the literature, providing valuable insights into the nutritional status of ambulatory cancer patients in Mexico. The scarcity of data on this population underscores the importance of our findings, which can inform future research and clinical practices. Enhancing the awareness and implementation of systematic nutritional screening and assessment in oncology is essential to improve patient outcomes and reduce the burden of malnutrition in this vulnerable population. Continued efforts to educate healthcare providers and integrate validated nutritional tools into standard care protocols are imperative for addressing malnutrition effectively in cancer patients.

We acknowledge some limitations in our study. The use of BMI as the sole marker of obesity is a limitation, as it does not differentiate between fat mass and muscle mass or account for fat distribution. Additionally, the lack of advanced tools for body composition analysis, such as bioelectrical impedance or DEXA, restricted the ability to assess parameters like muscle mass or visceral fat. Furthermore, the study did not account for potential confounding factors such as comorbidities, lifestyle habits, or socioeconomic status, which could influence nutritional outcomes. Also, the cross-sectional design of the study also limits the ability to establish causal relationships between nutritional status, obesity, and clinical outcomes. A longitudinal approach would provide deeper insights into how these factors interact over time. These limitations highlight the need for future research to incorporate more comprehensive assessments of body composition, consider confounding factors, and employ advanced statistical methods to better understand the complex interplay between cancer, nutrition, and obesity.

## 5. Conclusions

The study demonstrated significant variability in nutritional risk identification across screening tools, with the GLIM criteria identifying the highest prevalence of malnutrition due to its comprehensive approach, including disease burden and inflammation. The GLIM criteria and PG-SGA, along with reliable screening tests such as MUST, are essential for improving the nutritional care and outcomes of these patients. Screening tools showed lower sensitivity in detecting malnutrition among patients with obesity or overweight, highlighting the need for tailored methodologies.

HGS emerged as an effective functional and prognostic marker for malnutrition, particularly in gastrointestinal cancer patients. Additionally, 60.5% of ambulatory cancer patients were classified as overweight or obese, underscoring the importance of targeted nutritional interventions in oncology to address weight-related challenges and improve outcomes.

## Figures and Tables

**Table 1:** Patient characteristics.

Outpatient Cancer Patient Characteristics	
Variables	*n* (%)
Age	51.2 ± 13.2
Gender	
Male	111 (28)
Female	285 (72)
Tumor localization	
Breast	156 (39.4)
Colorectal	129 (32.6)
Other	63 (15.9)
Liver, pancreatic, and biliary tract	18 (4.5)
Gastric	18 (4.5)
Head and neck	12 (3)
Cancer Stage	
Stage I	99 (25)
Stage II	119 (30)
Stage III	99 (25)
Stage IV	79 (20)
Actual treatment	
chemotherapy and radiotherapy	24 (6.1)
chemotherapy	198 (50)
No current treatment	150 (37.9)
radiotherapy	9 (2.3)
Mean BMI	26.2 ± 5.06
BMI category	
Obesity	93 (23.5)
Overweight	147 (37.1)
Normal BMI	135 (34.1)
Undernutrition	21 (5.3)
Mean HGS	24.2 ± 8.4
HGS category	
Normal HGS	312 (78.8)
Low HGS	84 (21.2)
Weight loss in the last month	
No	228 (57.6)
Yes	168 (42.4)
%WL in the last month	
Moderate (<5)	147 (37.1)
Severe (>5)	21 (5.3)
Obesity/overweight in patients with weight loss	
No	60 (35.7)
Yes	108 (64.3)
Obesity/overweight in patients with moderate weight loss in the last month	
No	48 (32.7)
Yes	99 (67.3)
Obesity/overweight in patients with severe weight loss in the last month	
No	12 (57.1)
Yes	9 (42.9)
Oncological treatment in patients with Obesity/overweight	
No	105 (26.5)
Yes	134 (33.8)

BMI: body mass index; HGS: hand grip strength.

**Table 2:** Results between screening tools and nutritional assessment.

Test	Result	PG-SGA	GLIM	NRS-2002	MUST	MST	NUTRISCORE
PG-SGA	kappa	-	0.63	0.58	0.64	0.58	0.55
	Sensitivity %	-	93	73.3	73.5	68.6	94.1
	Specificity %	-	96.4	88.2	90.8	89.7	84.3
	PPV %	-	91.2	64.7	73.5	70.6	47.1
	NPV %	-	81.3	91.8	90.8	88.8	99
GLIM	kappa	0.63	-	0.53	0.68	0.61	0.32
	Sensitivity %	91.2	-	87.7	96	88.5	88.2
	Specificity %	81.3	-	77.4	82.9	81	70.1
	PPV %	62.8	-	53.3	66.2	62.8	30.4
	NPV %	74.2	-	77.2	74.2	73.4	87.1

PG-SGA: Patient-Generated Subjective Global Assessment; GLIM: Global Leadership Initiative on Malnutrition; NRS-2002: Nutritional Risk Screening-2002; MUST: Malnutrition Universal Screening Tool; PPV: positive predictive value; NPV: negative predictive value.

**Table 3:** Differences between screening tools in assessing malnutrition risk in patients with obesity or overweight.

Nutritional Assessment Test	Without Obesity/Overweight (*n* = 156)	Obesity/Overweight (*n* = 239)	Total (*n* = 395)	*p*-Value	OR (95% CI)
NRS-2002					
No nutritional risk	108 (69.2%)	198 (82.8%)	306 (77.5%)	0.002	0.4 (0.2–0.7)
Risk of malnutrition	48 (30.8%)	41 (17.2%)	89 (22.5%)		
MUST					
No nutritional risk	105 (67.3%)	188 (78.7%)	293 (74.2%)	0.012	0.5 (0.3–0.8)
Risk of malnutrition	51 (32.7%)	51 (21.3%)	102 (25.8%)		
MST					
No nutritional risk	114 (73.1%)	176 (73.6%)	290 (73.4%)	0.901	0.9 (0.6–1.5)
Risk of malnutrition	42 (26.9%)	63 (26.4%)	105 (26.6%)		
PG-SGA					
No nutritional risk	102 (65.4%)	191 (79.9%)	293 (74.2%)	0.001	0.4 (0.3–0.7)
Risk of malnutrition	54 (34.6%)	48 (20.1%)	102 (25.8%)		
NUTRISCORE					
No nutritional risk	129 (82.7%)	215 (90.0%)	344 (87.1%)	0.035	0.5 (0.2–0.9)
Risk of malnutrition	27 (17.3%)	24 (10.0%)	51 (12.9%)		
GLIM Criteria					
No malnourished	79 (50.6%)	168 (70.3%)	247 (62.5%)	<0.001	0.4 (0.2–0.6)
With malnutrition	77 (49.4%)	71 (29.7%)	148 (37.5%)		

NRS-2002: Nutritional Risk Screening; MUST: Malnutrition Universal Screening; MST: Malnutrition Screening Tool; PG-SGA: Patient-Generated Subjective Global Assessment; GLIM criteria: Global Leadership Initiative on Malnutrition.

## Data Availability

The data supporting the findings of this study are available from the corresponding author upon reasonable request.

## Abbreviations

The following abbreviations are used in this manuscript:

HGS	Handgrip strength
NS	Nutritional screening
ESPEN	European Society for Clinical Nutrition and Metabolism
MUST	Malnutrition Universal Screening Tool
MST	Malnutrition Screening Tool
PG-SGA	Patient-Generated Subjective Global Assessment
GLIM	Global Leadership Initiative on Malnutrition
BMI	Body mass index
SD	Standard deviation
